# Supplementary material for: Compromise or choose: shared movement decisions in wild vulturine guineafowl
Source: Commun Biol. 2024 Jan 13;7:95. doi: 10.1038/s42003-024-05782-w (PMC10787764; doi:10.1038/s42003-024-05782-w)
Supplement: Supplementary file 2 — Supplementary Material [file 42003_2024_5782_MOESM2_ESM.pdf]

Compromise or choose: Shared movement decisions in wild vulturine guineafowl

Danai Papageorgiou[1,2,3,4,5*], Brendah Nyaguthii[6,7,8], Damien R. Farine[1,2,8,9*]

1. University of Zurich, Department of Evolutionary Biology and Environmental Studies, Winterthurerstrasse 190, 8057 Zurich, Switzerland

2. Max Planck Institute of Animal Behavior, Department of Collective Behavior, Universitätsstraße 10, Konstanz, 78457, Germany

3. University of Konstanz, Department of Biology, Universitätsstraße 10, Konstanz, 78457, Germany

4. Kenya Wildlife Service, P.O. Box 40241-001000, Nairobi, Kenya

5. Wissenschaftskolleg zu Berlin, College for Life Sciences, Wallotstrasse 19, Berlin, 14193, Germany

6. University of Eldoret, School of Natural Resource Management, Department of Wildlife, 1125-30100 Eldoret, Kenya

7. Mpala Research Centre, P.O. Box 92, Nanyuki, 10400, Kenya.

8. National Museums of Kenya, Department of Ornithology, P.O. Box 40658-001000, Nairobi, Kenya

9. Australian National University, Division of Ecology and Evolution, Research School of Biology, 46 Sullivans Creek Road, Canberra, ACT 2600, Australia

*Correspondence: Danai Papageorgiou (danpapag@gmail.com) & Damien Farine (damien.farine@ieu.uzh.ch)

Supplementary Tables and Figures

Supplementary Table 1. GEE model results predicting the probability of following given the number of initiators, their level of agreement and their interaction (Group 2 has fewer data).

		**Estimate**	**Standard Error**	**Wald**	**P Value**
Group 1	Intercept	0.236	0.296	0.636	0.425
	Level of agreement	1.576	0.302	27.235	**<0.001**
	Number of Initiators	-0.438	0.066	43.327	**<0.001**
	Interaction: Level of agreement and number of Initiators	0.549	0.069	63.215	**<0.001**
Group 2	Intercept	0.297	1.197	0.061	0.804
	Level of agreement	0.773	1.212	0.407	0.524
	Number of Initiators	-0.777	0.406	3.657	0.056
	Interaction: Level of agreement and number of Initiators	1.011	0.415	5.931	**0.015**

Supplementary Table 2. Summary table on the extracted pulls, anchors and events per study group.

	Group 1		Group 2	
	Number	Percentage	Number	Percentage
Pulls	391,203		26,423	
Anchors	77,837		6,790	
All Events (concurrent pulls and anchors)	133,323		15,403	
Events with 1 initiator	52,446	37.492%	10,614	56.732%
Events with 2 initiators	24,004	17.160%	3,397	18.157%
Events with more than 2 initiators	63,437	45.349%	4,698	25,111%
Events with more than 2 initiators forming 1 cluster	11,114	17.520%	845	17.986%
Events with more than 2 initiators forming 2 clusters	23,568	37.152%	2,191	46.637%
Events with more than 2 initiators forming more than 2 clusters	28,755	45.329%	1,662	35.377%

Supplementary Table 3. The results of the non-linear least squares models applied on the data of each group separately, to test if in large levels of disagreement the guineafowl follow a majority rule. The only significant model that confirmed this hypothesis was for Group 1. Asym is the asymptote of the sigmoid curve, xmid is x-value of the infection point and scal is the angular coefficient of the tangent at point of inflection.

		**Estimate**	**Standard Error**	**T value**	**P Value**
Group 1	Asym	0.938	0.029	32.338	**<0.001**
	xmid	-1.427	0.676	- 2.111	**0.04**
	scal	5.927	0.521	11.370	**<0.001**
Group 2	Asym	1.138	0.173	6.560	**<0.001**
	xmid	0.228	1.537	0.150	0.885
	scal	3.902	1.078	3.620	**0.004**

Supplementary Table 4. Summary data from the two GPS tracked groups. The total number of birds includes the sub adults, which were less than 10 months old. Individuals less than ten months old have almost no influence in group decisions[1] and therefore we consider the number of adults as group size[2]. The number of tags refers to the number of GPS tags per group per month. Programming settings could differ from month to month with the tags working every fourth day or every day. The number of tagged individuals per group changed across the study period as some individuals got predated or lost their tags, respectively.

Group	Start Date	End Date	Total Number of Birds	Number of Adults	Number of Tags	Programming Settings
	21/02/18	23/03/18	25	24	24	every 4th day
	24/03/18	23/04/18	25	25	25	every 4th day
	24/04/18	24/05/18	24	23	23	every 4th day
	25/05/18	24/06/18	24	23	20	every 4th day
	25/06/18	25/07/18	22	21	19	every 4th day
Group 1	26/07/18	25/08/18	22	21	19	every 4th day
	26/08/18	25/09/18	23	23	23	every 4th day
	26/09/18	26/10/18	23	23	22	every 4th day
	27/10/18	26/11/18	23	23	22	every 4th day
	27/11/18	27/12/18	23	23	21	every 4th day
	28/12/18	19/01/19	23	23	19	every 4th day
	20/01/19	19/02/19	20	13	10	every 4th day
	20/02/19	22/03/19	20	13	11	every 4th day
	23/03/19	22/04/19	16	14	11	every day
	23/04/19	23/05/19	16	14	11	every day
Group 2	24/05/19	23/06/19	16	14	11	every day
	24/06/19	24/07/19	16	13	11	every 4th day
	25/07/19	24/08/19	20	14	10	every 4th day
	25/08/19	24/09/19	19	13	10	every 4th day
	25/09/19	25/10/19	19	13	10	every day

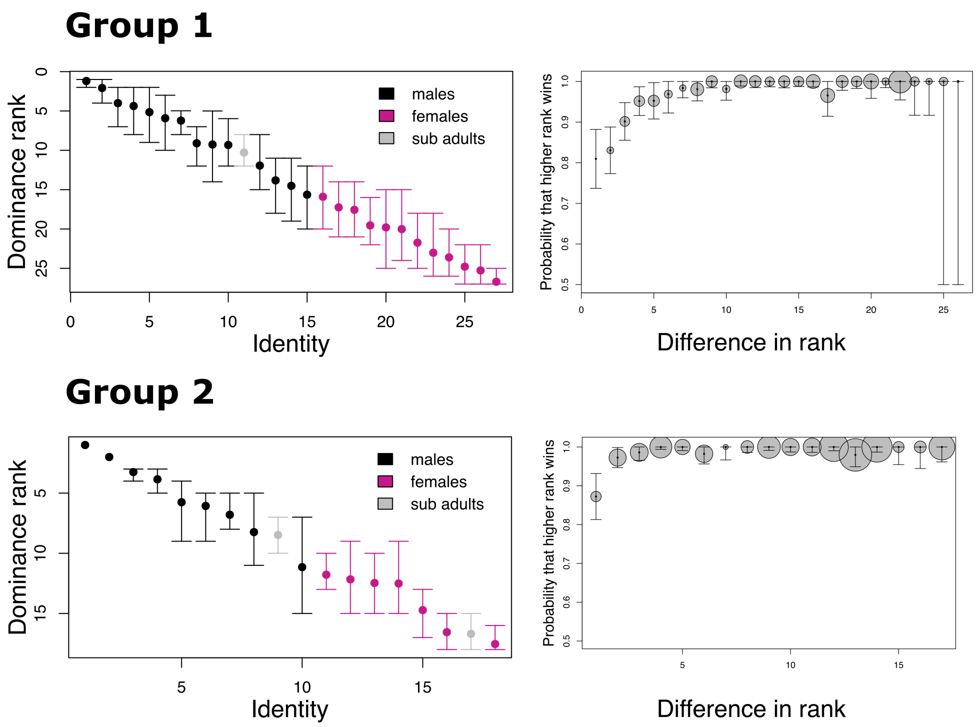

Supplementary Figure 1. Vulturine guineafowl maintain a steep dominance hierarchy with males being dominant over all females. These hierarchies remain stable for several months (Group 1: rs_{mean}=0.880, 95%CI=0.817–0.949, Group 2 rs_{mean}= 0.897, 95%CI=0.797–0.966, see Methods). Whiskers represent 95% confidence of rank estimates based on 1000 randomizations. Left panels: The dominance hierarchy of each of the two focal groups. Right panels: The probability of an individual to win an agonistic interaction, according to the difference in dominance.

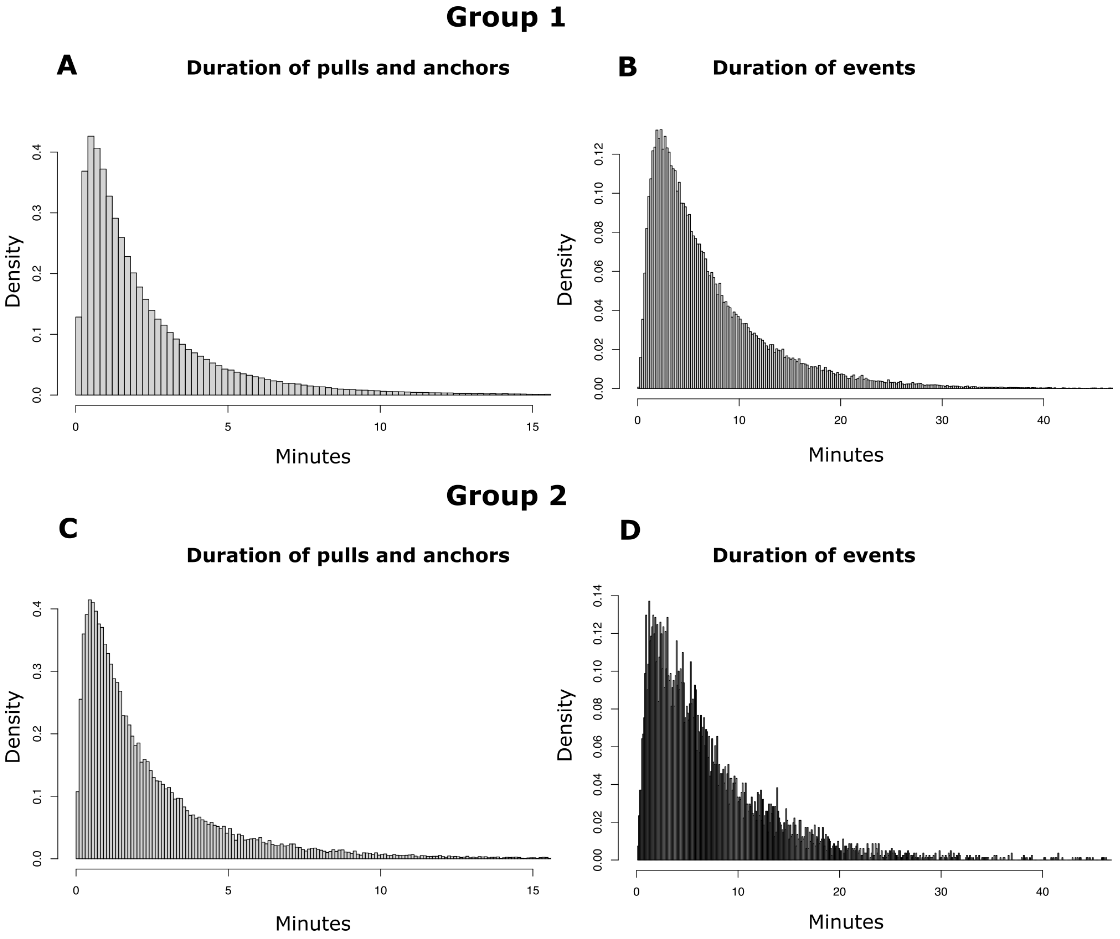

Supplementary Figure 2. The distribution of the time (in minutes) between (A, C) the start of an initiation by a leader and the moment that the leader ends the initiation and stops moving (before the pull or anchor takes place, see the second column in Figure 1; B, E, H, K). Panels B and D show the distribution of the time spent from the start (the first leader out of those participating in the event initiates movement) until the end of an event of multiple concurrent initiators (the follower of the event moves or the last leader comes back). Group's 1 data is depicted in A and B and Group's 2 in C and D.

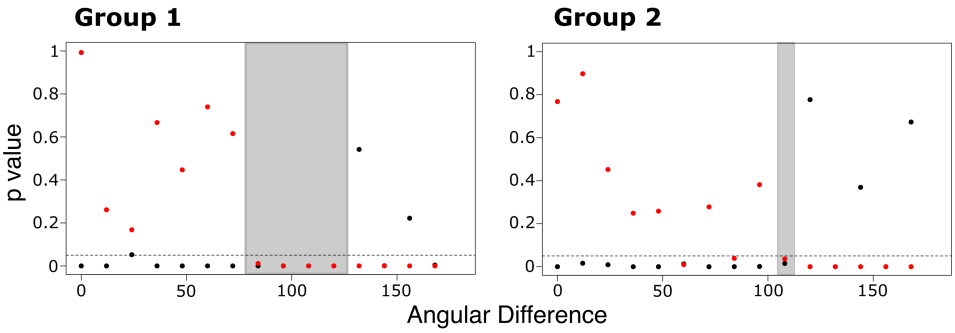

Supplementary Figure 3. Vulturine guineafowl transition from compromise to choose depending on the angular disagreement between initiators. We ran the converging-modes test (black dots) and the bimodality test (red dots) for Group 1 and for Group 2. We find that when angular difference between initiators is small, the distribution of directions taken by followers is significantly unimodal than expected by chance, suggesting that individuals are compromising between the two proposed directions. When the angular difference between initiators is above a critical threshold, follower directions were significantly bimodal, suggesting that followers chose one direction or the other.

Supplementary Note 1: Supplementary Analyses

Here we present all the results of the main text and of the Supplementary Material after applying a threshold of 80% of group members' tags collecting data at the same time. We extracted 378,410 leader-follower cases from the two focal groups. For Group 1, which had the most data, all results were replicated. For Group 2, which had a much smaller dataset (see Supplementary Table 6), some of the results were not significant at the 80% threshold.

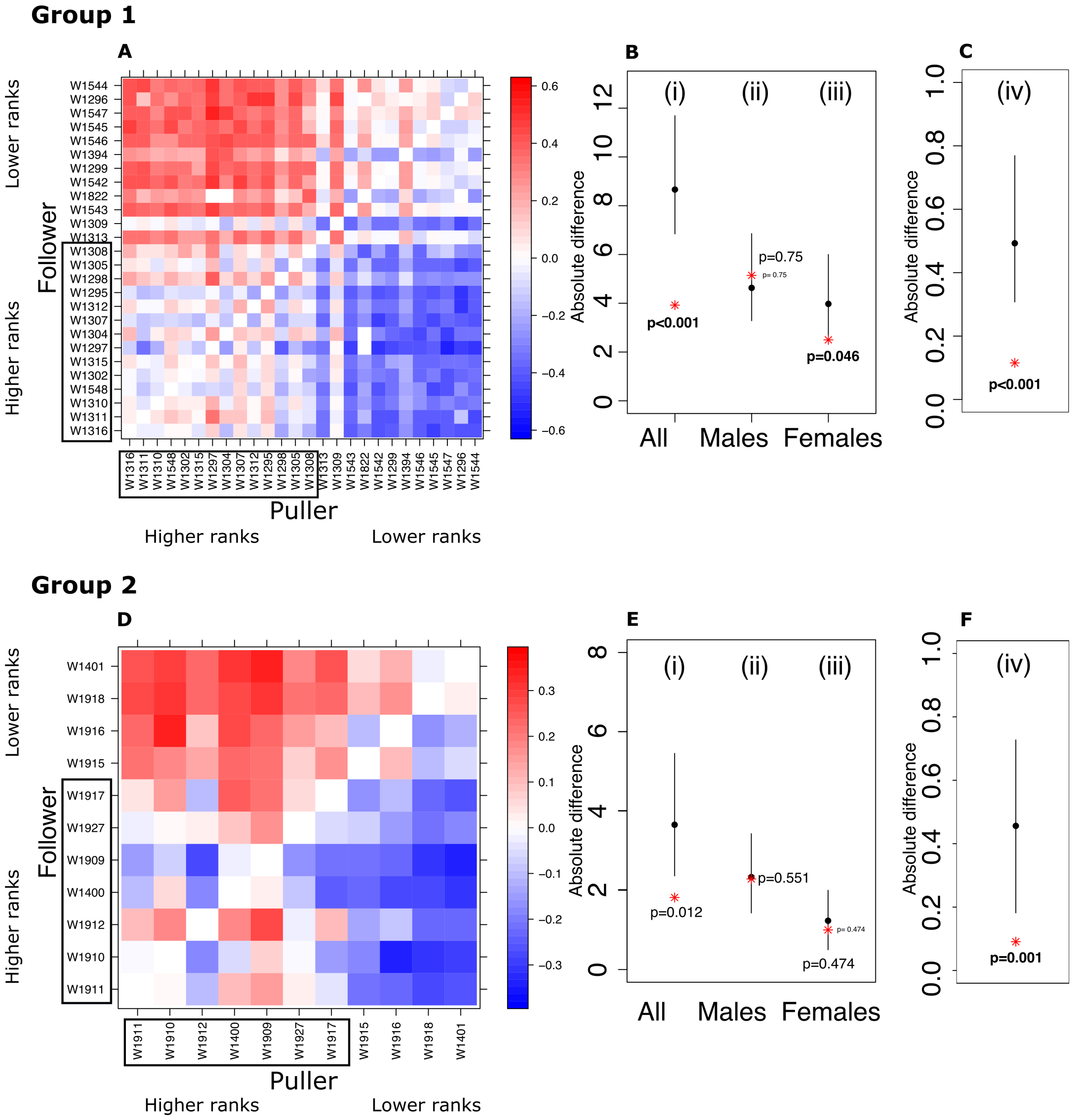

Supplementary Figure 4. Replicate of Figure 2 when a minimum of 80% of group members were collecting data.

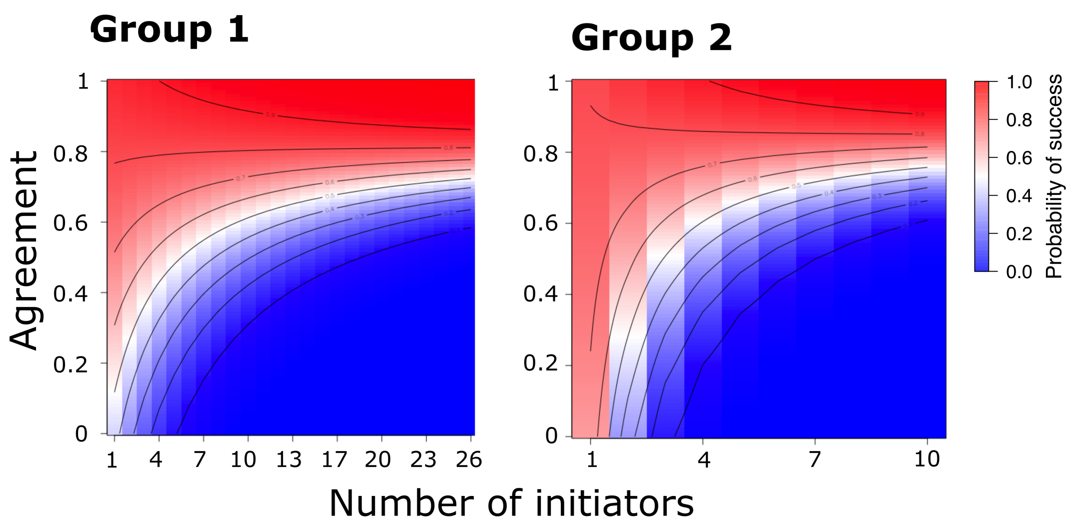

Supplementary Figure 5. Replicate of Figure 4 when a minimum of 80% of group members were collecting data.

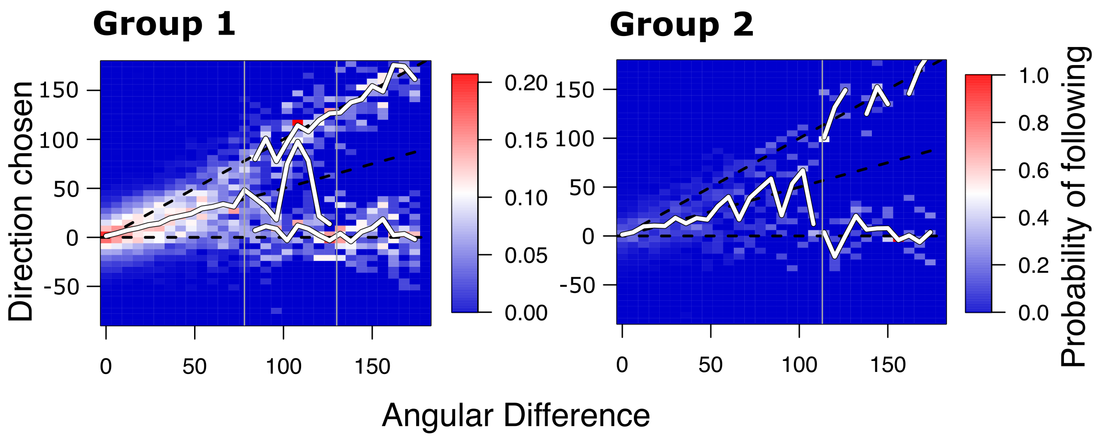

Supplementary Figure 6. Replicate of Figure 5 when a minimum of 80% of group members were collecting data.

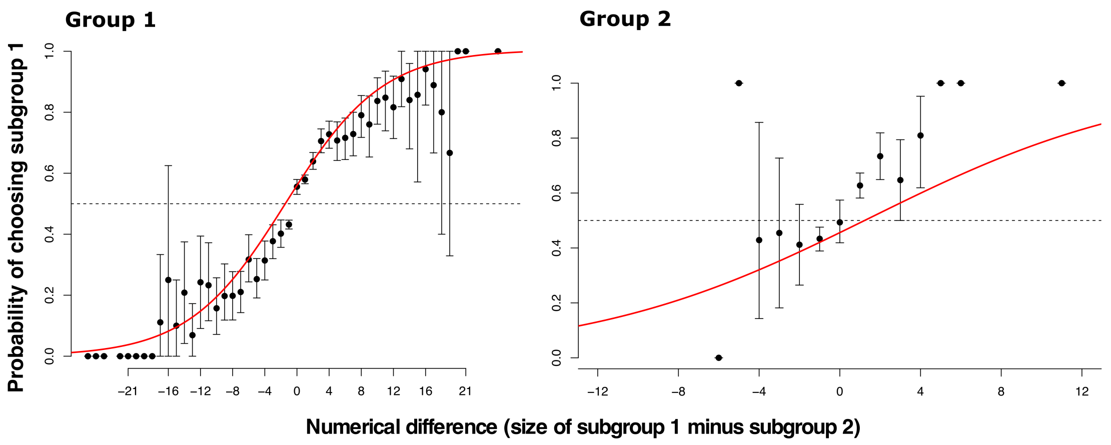

Supplementary Figure 7. Replicate of Figure 6 when a minimum of 80% of group members were collecting data.

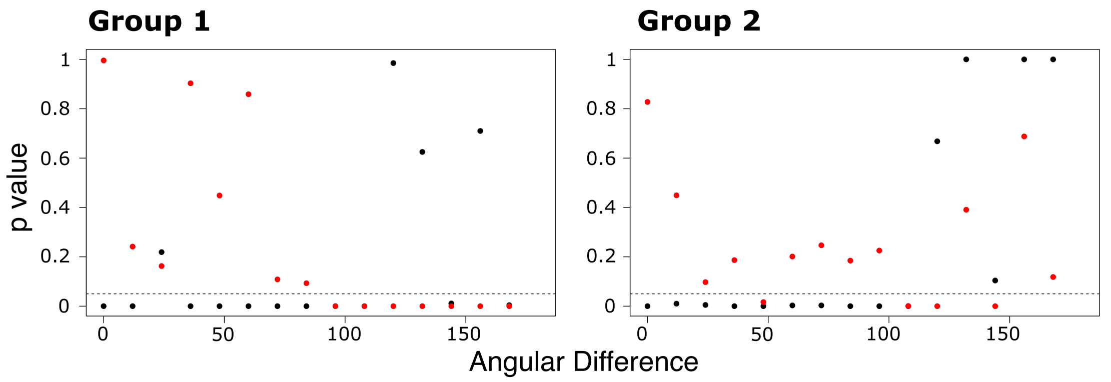

Supplementary Figure 8. Replicate of Supplementary Figure 3 when a minimum of 80% of group members were collecting data.

Supplementary Table 5. Replication of Supplementary Table 1 when a minimum of 80% of group members were collecting data.

		Estimate	Standard Error	Wald	P Value
Group 1	Intercept	0.216	0.363	0.353	0.552
	Level of agreement	1.564	0.370	17.877	**<0.001**
	Number of Initiators	-0.466	0.080	34.022	**<0.001**
	Level of agreement: Number of Initiators	0.570	0.083	47.349	**<0.001**
Group 2	Intercept	1.981	1.073	3.409	0.065
	Level of agreement	-0.780	1.084	0.518	0.472
	Number of Initiators	-1.321	0.340	15.071	**<0.001**
	Level of agreement: Number of Initiators	1.560	0.348	20.062	**<0.001**

Supplementary Table 6. Replication of Supplementary Table 2 when a minimum of 80% of group members were collecting data.

	**G1**		**G2**	
	Number	Percentage	Number	Percentage
Pulls	293,755		19,428	
Anchors	60,869		4,358	
All Events (concurrent pulls and anchors)	98,397		10,934	
Events with 1 initiator	77,979	55.74%	13,166	70.37%
Events with 2initiators	17,037	12.18%	2,321	12.41%
Events with more than 2 initiators	44,871	32.08%	3,222	17%
Events with more than 2 initiators forming 1 cluster	8,026	17.89%	573	17.78%
Events with more than 2 initiators forming 2 clusters	16,720	37.26%	1,483	46.03%
Events with more than 2 initiators forming more than 2 clusters	20,125	44.85%	1,166	36.19%

Supplementary Table 7. Replication of Supplementary Table 3 when a minimum of 80% of group members were collecting data.

		Estimate	Standard Error	T value	P Value
G1	Asym	0.937	0.030	30.890	**<0.001**
	Xmid	-1.386	0.712	-1.950	0.058
	Scal	6.040	0.545	11.080	**<0.001**
G2	Asym	1.138	0.174	6.561	**<0.001**
	Xmid	0.226	1.537	0.148	0.885
	Scal	3.902	1.078	3.620	**0.004**

Supplementary Table 8. We re-run here the GEE models of Supplementary Table 1. Independent variables predicting the probability of following were the number of initiators, their level of agreement and their interaction (Group 2 has fewer data), as well as the proportion of tags that were operating when the initiation took place and the distance between the leader and the follower.

		Estimate	Standard Error	Wald	P Value
Group 1	Intercept	-0.039	0.300	0.017	0.898
	Level of agreement	1.605	0.305	27.691	**<0.001**
	Number of Initiators	-0.435	0.067	42.281	**<0.001**
	Interaction: Level of agreement and number of Initiators	0.551	0.069	62.824	**<0.001**
	Proportion of tags working	1.509	0.116	168.742	**<0.001**
	Distance between leader and follower	0.006	0.003	5.042	**0.025**
Group 2	Intercept	0.432	1.199	0.130	0.719
	Level of agreement	0.701	1.212	0.335	0.563
	Number of Initiators	-0.787	0.406	3.760	0.053
	Interaction: Level of agreement and number of Initiators	1.013	0.415	5.973	**0.015**
	Proportion of tags working	-0.867	0.202	18.397	**<0.001**
	Distance between leader and follower	0.036	0.007	31.163	**<0.001**

Supplementary References

1. Papageorgiou, D. & Farine, D. R. Shared decision-making allows subordinates to lead when dominants monopolize resources. *Sci. Adv.* **6**, eaba5881 (2020).
2. Papageorgiou, D. & Farine, D. R. Group size and composition influence collective movement in a highly social terrestrial bird. *Elife* **9**, 1–16 (2020).
