# Supplementary material for: Compromise or choose: shared movement decisions in wild vulturine guineafowl
Source: Commun Biol. 2024 Jan 13;7:95. doi: 10.1038/s42003-024-05782-w (PMC10787764; doi:10.1038/s42003-024-05782-w)
Supplement: Supplementary file 3 — Description of Additional Supplementary Files [file 42003_2024_5782_MOESM3_ESM.pdf]

Description of Additional Supplementary Files

File name: Supplementary Movie 1
Description: Movement of Group 1 on 26.03.2018 at 07:22

File name: Supplementary Movie 2
Description: Movement of Group 1 on 26.03.2018 at 09:27

File name: Supplementary Movie 3
Description: Movement of Group 1 on 26.03.2018 at 10:09

File name: Supplementary Movie 4
Description: Movement of Group 2 on 24.01.2019 at 10:01

File name: Supplementary Movie 5
Description: Movement of Group 2 on 01.02.2019 at 15:16

File name: Supplementary Movie 6
Description: Movement of Group 2 on 15.07.2019 at 10:08
